# Association Between Availability of Fruits and Vegetables in Neighborhood Food Stores and Weight Among Residents of Low-Income Urban Public Housing: Cross-Sectional Study

**DOI:** 10.2196/81581

**Published:** 2026-02-09

**Authors:** Robert Leung, Allison Frank, Lynsie R Ranker, Jennifer Murillo, Kevin J Lane, Zachary T Popp, John Kane, Ziming Xuan, Belinda Borrelli, Lisa M Quintiliani

**Affiliations:** 1 Veterans Administration Boston Healthcare System, Boston, MA, United States; 2 Department of Community Health Sciences, Boston University School of Public Health, Boston, MA, United States; 3 Division of General Internal Medicine, Tufts Medical Center, Boston, MA, United States; 4 Department of Environmental Health Sciences, Boston University School of Public Health, Boston, MA, United States; 5 Boston Housing Authority, Boston, MA, United States; 6 Center for Behavioral Science Research, Boston University Henry M Goldman School of Dental Medicine, Boston, MA, United States; 7 Department of Medicine, Tufts University, 185 Harrison Ave, Center Building, 2nd Floor, Boston, MA, 02111, United States, 1 617-636-5346, 1 617-636-8568

**Keywords:** food environment, fruits, vegetables, low-income, weight

## Abstract

This cross-sectional study examined the presence of food stores and availability of fruits and vegetables in food stores with weight among urban public housing residents. While there was no association between average number of fruits or vegetables in food stores and weight, there were positive associations between number of convenience stores and weight and between number of general merchandise stores and weight.

## Introduction

Though food environments are recognized as determinants of socioeconomic diet–related health disparities, there is less evidence delineating specific elements of food environments that contribute to bodyweight [1]. Previous literature is limited by variability in measurement approaches and a lack of capturing multiple food retail environments [1]. Among adults, a systematic review or meta-analysis assessing impact of food environments on obesity included only two studies with general merchandise stores as part of their analyses [1]. Furthermore, much research about general merchandise stores focuses on rural areas and children or adolescent populations, prompting evaluation among adults in urban areas [2,3]. This study’s objective was to examine associations between two measures of food access: (1) presence of food stores (overall and by store types) and (2) availability of fruits and vegetables with weight at study enrollment among residents of urban public housing developments participating in a clinical trial for weight management [4].

## Methods

### Design

This is a cross-sectional analysis of baseline data from a clinical trial (Table 1) [1,4] along with neighborhood food store audit data collected by research personnel.

**Table 1.** Description of the Path to Health Behavioral Clinical Trial for Weight Management (Recruitment period: 2021-2024; ClinicalTrials.gov #NCT04852042) [4]

Randomized groups	• Participants were randomized to: º Assessment only control (Control); º mHealth weight management intervention (Text messaging program and weight monitoring via digital scale) (mHealth only); º mHealth intervention plus motivational interviewing-based behavioral counseling delivered by a community health worker (mHealth+CHW)
Primary objective	• To evaluate if the mHealth+CHW intervention demonstrates efficacy for weight loss • Primary hypothesis: Both mHealth+CHW and mHealth only will outperform assessment only control and mHealth+CHW will outperform mHealth only in terms of weight loss at 12 months.
Eligibility criteria	• Trial inclusion criteria were: age >=18 years old; residents of Boston public housing; BMI >=27.0, and read/write English or Spanish (full criteria available [4]).
Study setting	• Our study setting consisted of family-designated housing developments (n=17) in the City of Boston managed by the Boston Housing Authority (n=9) or private property management companies (n=8) which receive subsidies for renting to low-income households.
Baseline characteristics	• Among 286 participants, 83.2% were female, 70.6% were Hispanic, 63.6% with up to high school education, with mean age of 53.6 (SD 14) years and mean weight of 188.1 (SD 42.9) pounds.

### Measures

#### Weight

Research personnel measured participant (n=286) weight via scale during in-person, in-home baseline study visits [4].

#### Food Store Audit Instrument

Pairs of research personnel conducted in-store audits using the Food Environment Audit for Diverse Neighborhoods to identify food availability [5].

### Data Analysis

Food access measures (number of food stores; total count of available fruits and vegetables or audited food store) were assigned to individual participants in the clinical trial, based on their public housing development of residence. We used separate generalized linear models examining associations between each access metric and participant weight at baseline. Specifically, we used generalized estimating equations to account for clustering of participants within housing developments (adjusted for resident age, gender, Hispanic ethnicity, and education). The model coefficient represents the difference in weight at baseline enrollment with a one-unit increase in count (ie, one additional store, or one additional fruit and vegetable available). Additional models adjusting for height were conducted (Multimedia Appendix 1). See Table 2 footnotes for details.

**Table 2.** Descriptive information and associations between food access measures (number of food stores and availability of fruits and vegetables) and baseline weight (n=286 participants).

Descriptive information	Values
Total number of food stores within 1 mile, mean (SD)	23.6 (13.9)
Supermarkets/other grocery stores	11.1 (7.1)
General merchandise/dollar stores	2.5 (1.7)
Convenience stores	10.1 (6.5)
Fruits (fresh, frozen, canned, juice), vegetables (fresh, frozen, canned, juice), and beans (canned, dried) available in all audited food stores (n=31**)**	
At least one type of fruits & vegetables available, n (%)	30 (96.8)
10 or more types of fruits & vegetables available, n (%)	24 (77.4)
Number of fruits & vegetables, mean (SD)	32.6 (39.2)
Number of stores within 1 mile, unadjusted difference in weight (95% CI)	0.34 (-0.06, 0.73)
Supermarkets/other grocery stores	0.03 (-1.06, 1.12)
General merchandise/dollar stores^a^	3.11 (0.45, 5.76)
Convenience stores	1.12 (0.34, 1.91)
Number of stores^a^ within 1 mile, adjusted^b^ difference in weight (95% CI)	0.33 (−0.05, 0.71)
Supermarkets/other grocery stores	0.25 (−0.51, 1.01)
General merchandise/dollar stores^a^	3.08 (1.31, 4.85)
Convenience stores	0.87 (0.02, 1.72)
Number of fruits and vegetables,^c^ unadjusted difference in weight (95% CI)	0.09 (-0.36, 0.53)
Supermarkets/other grocery stores	0.02 (-0.13, 0.17)
General merchandise/dollar stores^d^	0.05 (-0.38, 0.49)
Convenience stores	0.58 (-0.88, 1.41)
Number of fruits and vegetables^c^ (Adjusted^b^ difference in weight (95% CI)	0.08 (−0.26, 0.41)
Supermarkets/other grocery stores	0.02 (−0.11, 0.14)
General merchandise/dollar stores^d^	0.10 (−0.22, 0.41)
Convenience stores	0.30 (−0.40, 1.00)

a *Identification of Food Stores*: We used a commercial database called Data Axle Reference Solutions which contained location and business data on all U.S. businesses. Addresses for public housing developments and food stores were geocoded. ArcGIS Pro version 3.0 was used to model walking distance for one mile around public housing developments where participants lived. We chose one mile in order to capture a wide range of available food stores [6]. We identified three food store types using the North American Industry Classification Codes [NAICS], specifically including stores coded as: grocery stores (NAICS: 445110) retailing canned and frozen foods, fresh fruits and vegetables, and fresh and prepared meats, fish, and poultry; convenience stores (NAICS: 445120) retailing a limited line of goods including milk, bread, soda, and snacks; and all other general merchandise stores (NAICS: 452319) retailing general merchandise including dry goods as well as other merchandise such as apparel, housewares, and home furnishings. These food stores were categorized according to NAICS criteria. Our data manager used a random number generator to select one store to be audited from each food store category corresponding to each public housing development included in the Path to Health trial. We also measured food access via the total number of stores (and number within each food store category) within the walking buffer. Prior to conducting the audits, research staff conducted a spot check that actual store matched the expected store. In the audit, fruits and vegetables were counted as present or not present, instead of the actual number of individual fruits and vegetables.

b Separate generalized linear models adjusted for resident age, gender, Hispanic ethnicity, and education accounting for clustering within housing developments.

c Data represents food audits at eleven food store locations: Three groups of two public housing developments and one group of four public housing developments located within one mile of each other were combined into a single audit location due to proximity and overlapping identification of food stores. This process resulted in eleven unique food audit locations. From 11 audit locations (which corresponded to 17 housing developments), we audited 31 food stores (2 housing developments did not have a general merchandise store available for auditing). The total count of unique fruits, vegetables, and bean types available was created for each audited store and then these counts were averaged within each audit location.

d n=236 participants, due to the exclusion of two housing developments that did not have a general merchandise store available for auditing.

### Ethical Considerations

Boston University Medical Campus Institutional Review Board approved this analysis (H-41590). The original written consent process covered this analysis without additional consent. Privacy was ensured by deidentifying data with code numbers. Participants were paid US $50 for completing baseline assessment activities.

## Results

There was no association between overall number of food stores and baseline weight nor between average number of fruits and vegetables in audited food store locations and baseline weight (adjusted difference in weight: 0.33, [95% CI −0.05,0.71 and 0.08, 95% CI: −0.26, 0.41 pounds, respectively). When food store type was examined, there were positive associations between number of convenience stores and baseline weight and between number of general merchandise stores and baseline weight (adjusted difference in weight: 0.87, 95% CI: 0.02, 1.72 and 3.08, 95% CI: 1.31, 4.85 pounds, respectively) (Table 2). Additional adjustment for height resulted in associations in a similar direction, with a small attenuation in magnitude (Multimedia Appendix 1).

## Discussion

The number of convenience stores and general merchandise stores within a one-mile walking distance radius of public housing developments were associated with higher baseline weight, after adjusting for potential confounding factors. Unlike findings from systematic reviews or meta-analyses [1], we did not observe associations with number of grocery stores and baseline weight or availability of fruits and vegetables at audited food stores and baseline weight. Weight status is multifactorial and therefore, auditing a larger variety of foods may have been necessary to observe an association with weight. Others also noted lack of associations between new grocery stores and healthful eating among public housing residents [7]. Overall, neighborhood environment deprivation factors like lack of safe physical activity areas can all contribute to adverse health behaviors and outcomes.

Dollar stores are the fastest growing food retail type by share of household expenditure in the United States [3], raising concerns about nutrition and health-related outcomes [8] and impact on local businesses, staffing shortages, and overall cleanliness [9]. Municipalities have taken policy actions to address concerns including: moratoriums (prohibit new store openings for specified period) and conditional use ordinances (require local government to assess how proposed store aligns with certain conditions) [9]. Obtaining perspectives from key constituents and forming partnerships (eg, community members or policy makers workshops) can lead to creation of fair policies for equitable benefit [10].

This study benefited from a standardized validated audit data collection instrument and included diverse food stores. Limitations included: lack of consideration for sales volume; audits represented snapshots of food available that particular day or season; and inclusion of clinical trial participants potentially limited generalizability.

Our study suggests cross-sectional relationships between presence of convenience stores and general merchandise stores and higher weight among residents that were overweight or with obesity living in nearby public housing developments; findings may inform design of community-level food store policies.

## Supplementary material

10.2196/81581 Multimedia Appendix 1 Descriptive information and associations between food access measures (number of food stores and availability of fruits and vegetables) and baseline weight.

## References

[1] Pineda E, Stockton J, Scholes S, Lassale C, Mindell JS (2024). Food environment and obesity: a systematic review and meta-analysis. BMJ Nutr Prev Health.

[2] Gorski Findling MT, Wolfson JA, Rimm EB, Bleich SN (2018). Differences in the neighborhood retail food environment and obesity among us children and adolescents by SNAP Participation. Obesity (Silver Spring).

[3] Feng W, Page ET, Cash SB (2023). Dollar stores and food access for rural households in the United States, 2008‒2020. Am J Public Health.

[4] Solar C, Nansubuga A, Murillo J (2022). Mobile health plus community health worker support for weight management among public housing residents (Path to Health): a randomized controlled trial protocol. Contemp Clin Trials.

[5] Izumi BT, Zenk SN, Schulz AJ (2012). Inter-rater reliability of the food environment audit for diverse neighborhoods (FEAD-N). J Urban Health.

[6] Access to affordable and nutritious food: updated estimates of distance to supermarkets using 2010 data. Economic Research Service.

[7] Datar A, Liu Y, Shier V (2025). Supermarket opening in an urban, low-income community was not found to be associated with improvements in dietary outcomes for most residents in the first year. J Acad Nutr Diet.

[8] Sundermeir SM, Matsuzaki M, Zhang A, Obi JC, Gittelsohn J, Winkler MR (2025). Characterizing the consumer food environment of dollar stores and exploring differences by neighborhood racial composition. J Nutr Educ Behav.

[9] Sundermeir SM, Santos SR, Lewis EC (2024). Dollar store policy opportunities in Baltimore City: community member and policy maker perspectives. Front Nutr.

[10] Langellier BA, Argibay S, Henson RM (2024). Participatory systems thinking to elucidate drivers of food access and diet disparities among minoritized urban populations. J Urban Health.

