# Supplementary material for: Association Between Availability of Fruits and Vegetables in Neighborhood Food Stores and Weight Among Residents of Low-Income Urban Public Housing: Cross-Sectional Study
Source: JMIR Form Res. 2026 Feb 9;10:e81581. doi: 10.2196/81581 (PMC12885180; doi:10.2196/81581)
Supplement: Multimedia Appendix 1 [file formative-v10-e81581-s001.docx]

Supplemental Table. Descriptive information and associations between food access measures (number of food stores and availability of fruits and vegetables) and baseline weight (n=286 participants)

|  | **Adjusted^a^ difference in weight, accounting for height (95% CI)** |
| --- | --- |
| **Number of stores^b^ within 1 mile** | 0.16 (-0.15, 0.47) |
| Supermarkets/other grocery stores | -0.11 (-0.78, 0.56) |
| General merchandise/dollar stores^b^ | 1.97 (-0.47, 4.41) |
| Convenience stores | 0.63 (-0.03, 1.29) |
| **Number of fruits and vegetables**^c^ | 0.13 (-0.23, 0.49) |
| Supermarkets/other grocery stores | 0.03 (-0.09, 0.15) |
| General merchandise/dollar stores^d^ | 0.22 (-0.13, 0.58) |
| Convenience stores | 0.33 (-0.41, 1.07) |

^a^Separate generalized linear models adjusted for resident age, gender, Hispanic ethnicity, education, and height accounting for clustering within housing developments.

^b^*Identification of Food Stores.* We used a commercial database called Data Axle Reference Solutions which contained location and business data on all U.S. businesses. Addresses for public housing developments and food stores were geocoded. ArcGIS Pro version 3.0 was used to model walking distance for one mile around public housing developments where participants lived. We chose one mile in order to capture a wide range of available food stores.[6] We identified three food store types using the North American Industry Classification Codes [NAICS], specifically including stores coded as: grocery stores (NAICS: 445110) retailing canned and frozen foods, fresh fruits and vegetables, and fresh and prepared meats, fish, and poultry; convenience stores (NAICS: 445120) retailing a limited line of goods including milk, bread, soda, and snacks; and other food stores (NAICS: 452311) retailing fresh fruits, vegetables, dairy products, meats, and other perishable groceries as well as other merchandise such as apparel and appliances. These food stores were categorized according to NAICS criteria. Our data manager used a random number generator to select one store to be audited from each food store category corresponding to each public housing development included in the Path to Health trial. We also measured food access via the total number of stores (and number within each food store category) within the walking buffer. Prior to conducting the audits, research staff conducted a spot check that said store matched the expected store. In the audit, fruits and vegetables were counted as present or not present, instead of the actual number of individual fruits and vegetables.

^c^Data represents food audits at eleven food store locations: Three groups of two public housing developments and one group of four public housing developments located within one mile of each other were combined into a single audit location due to proximity and overlapping identification of food stores. This process resulted in eleven unique food audit locations. From 11 audit locations (which corresponded to 17 housing developments), we audited 31 food stores (2 housing developments did not have a general merchandise store available for auditing). The total count of unique fruits, vegetables, and bean types available was created for each audited store and then these counts were averaged within each audit location.

^d^n=236 participants, due to the exclusion of two housing developments that did not have a general merchandise store available for auditing.
